# Supplementary material for: Etiologies and 12-month mortality in patients with isolated involuntary weight loss at a rapid diagnostic unit
Source: PLoS One. 2021 Sep 23;16(9):e0257752. doi: 10.1371/journal.pone.0257752 (PMC8459999; doi:10.1371/journal.pone.0257752)
Supplement: S1 File — (DOCX) [file pone.0257752.s001.docx]

**S1 File. Criteria for referral to the RDU in patients over the age of 18.**

| **Signs and symptoms warranting referral to the RDU** |
| --- |
| Involuntary weight loss |
| Jaundice or notable alteration of liver enzymes |
| Iron deficiency anemia |
| Hemoglobin <10g/dl |
| Significantly enlarged lymph nodes |
| Visceromegaly |
| Ascites |
| Suspicion of bone or lung metastasis |
| Dysphagia (last 6 months) |
| Gastroscopic findings suggestive of malignancy |
| Suspicion of systemic disease |
| Radiological image (X-ray, abdomen ultrasound and thoracic or abdomen CT scan) suggestive of malignancy |
| Guide signs or symptoms of colorectal cancer |
| Iron deficiency anemia of unknown origin |
| Rectal bleeding |
| Intestinal transit disorders (>1 month) |
| Diarrhea < 6 months |
| Intestinal subocclusion crises |
| Abdominal mass |
| Suspicion of malignant hepatomegaly |
| Tenesmus/suspicious rectal examination |
| Colonoscopic findings suggestive of malignancy |
