# Supplementary material for: Etiologies and 12-month mortality in patients with isolated involuntary weight loss at a rapid diagnostic unit
Source: PLoS One. 2021 Sep 23;16(9):e0257752. doi: 10.1371/journal.pone.0257752 (PMC8459999; doi:10.1371/journal.pone.0257752)
Supplement: Attachment — Submitted filename: Review.docx [file pone.0257752.s002.docx]

In this paper the authors reviewed retrospectively patients presented to the rapid diagnostic unit (RDU) for isolated involuntary weight loss (IWL) in a period between 2005 and 2013. The study was a single-center one. Follow up was performed at 1 year. From the 1592 identified patients, 791 (49.7%) met the criteria for enrollment into the study, which is the largest study to date for this group of patients. The main results of the study are that non-malignant organic diseases was the cause for IWL in 44.5%, psychiatric disorders in 29.0%, malignant diseases in 23.6%, and unknown causes in 3.2%. Mortality at 12 months was 18.6% overall, which was much higher in the malignant group 61.1% (95%CI: 54.2-68.2). The authors conclude that malignancy should be ruled out during the first visit for patients presenting with IWL.

The manuscript is pretty well written, graphs illustrative, and its findings could be of some interest. The results of the paper are however not novel, add little to the existing data, but the cohort is large, though retrospective. The discussion section could have been constructed in a more attractive way.

Besides, I think there are some issues with this paper:

Major:

1. The main drawbacks are that it is a single center study with retrospective design. Also, the studied period ended with more than 7 years ago, which is of some concern. Why wasn’t the study period ended sooner, such as in 2019, with follow-up ending in 2020?
2. Materials and methods: the authors state that the study included “patients visited at the RDU between January 2005 and December 2013”, and that the “follow-up of the last patient was done in December 2014”. However, below, the authors state that the follow-up was of 6 months, which is different than the 1-year interval presented above. Please explain the difference.
3. Materials and methods: Please explain why patients with mild or chronic symptoms represented exclusion criteria for being referred to the RDU, because it is not clear, and because I suppose that most of the enrolled patients had some mild and chronic symptoms.
4. Results: Please review the fact that “mean weight loss in the past 12 months was 8.3 Kg (SD 4.7)”, because this would mean that some of the patients lost less than 5kg, which is a non-diagnostic criterion for IWL. See also the data in table 1, in which SD seems to be discordant between the columns of the amounts of weight loss.
5. In the conclusion of the paper, the authors state that “computed tomography is the most profitable complementary test to be performed in addition to complete anamnesis and blood tests for a good diagnostic approximation.” However, there is no discussion throughout the paper about the value of complete anamnesis and of blood tests in establishing the diagnosis in this cohort of patients.

Minor:

1. Abstract: please replace “Mortality at 12 months was higher…” with “Mortality at 12 months was **much** higher…”, as the differences are quite important between the two groups.
2. Introduction: please replace “ambulatory rapid diagnostic unit (RDU) allow us…” by “ambulatory rapid diagnostic unit**s** (RDU**s**) allow us…”. Also, “In these RDU…” by “In these RDU**s**…”.
3. Introduction: please review the expression “organic or malignant 32 to 51%”; it could be organic or **non-**malignant, and there could be a mistake.
4. Material and methods: “Ethical Committee number CEIC 15/16” should be integrated into a sentence.
5. Material and methods: it is not clear what it is meant by “serious rhythm deposition changes”. Please rephrase or explain better.
6. Material and methods: The comma after “lymph nodes” should be deleted.
7. Table 1: change “UDR” to “RDU”.
8. Table 2: please rephrase “ESR, GGT i AP”.
9. Table 2: if the values between square brackets represent percentile 25-percentile 75, please state as such: **[**percentile 25-percentil**e** 75**]**, and not “(percentile 25-percentil75)”.
10. Results: please rephrase “11.9% of thoracoabdominal CTs showed images suspicious for malignancy (which proved to be false positive…” to “11.9% of thoracoabdominal CTs showed images **considered** suspicious for malignancy (which proved to be false positive…”.
11. Results: please add results of performing upper gastrointestinal endoscopy, not only colonoscopy, especially as below the authors state that “In the neoplastic group, 50% of gastroscopies… were histologically confirmed as malignant.”
12. Table 4: delete repeated word (benign). What do you mean by significant when referring to colon polyps?
13. Table 4: what does enolism stand for?
14. Results: please put “per cent” together.
15. Discussion: please rephrase: “Computed tomography is the most profitable complementary test”; profitable does not seem appropriate for this instance.
16. Figure 3: what do the figures below the graph represent (no. of patients at risk)? Is it the number of patients followed-up at the specific time intervals? It is not clear.
