# Supplementary material for: Etiologies and 12-month mortality in patients with isolated involuntary weight loss at a rapid diagnostic unit
Source: PLoS One. 2021 Sep 23;16(9):e0257752. doi: 10.1371/journal.pone.0257752 (PMC8459999; doi:10.1371/journal.pone.0257752)
Supplement: Attachment — Submitted filename: Althaia R2 Response to Reviewers.docx [file pone.0257752.s004.docx]

Dear Editor and Reviewers,

The authors are grateful for your revision of the draft manuscript **PONE-D-20-05824R1**. Your comments are highly appreciated and have helped us to improve the quality of the text.

Please find below a point-by-point response to all the reviewers’ comments, along with the amended version of the document with the new text presented in red, which we are resubmitting for consideration and possible publication.

We look forward to hearing from you.

Yours faithfully,

Jordi Aligué, MD, PhD

Althaia Xarxa Assistencial Universitària de Manresa

**Point by point response to the reviewers’ comments**

Ref: **PONE-D-20-05824R1**

**“Etiologies and 12-month mortality in patients with isolated involuntary weight loss at a rapid diagnostic unit”**

**Reviewer #2:** There are three minor comments:

**MINOR CHANGES:**

**Specific minor comment #1**

- in the material and methods section, line 111, please change "it was not able" to "it was not possible".

**Response:** Modified

**Specific minor comment #2**

- please review again the SD (standard deviations) for the amount of weight loss (kg) in Table 1 (in the first column SD in 4.7, while in all the others it is 0.3 and 0.8, which is somehow odd)

**Response:** We have detected a transcription error in the standard deviations of the variables age, weight loss (kg) previous 12 months, daily alcohol consumption (g/day) and Charlson Comorbidity Index. We have amended the values in the table 1. We apologize for the error.

**Specific minor comment #3**

- in the discussion section, lines 58-59, please amend this formulation, since gastric, colonic and pancreatic are digestive cancers.

**Response:** Modified. The percentage of digestive cancers is calculated over the total of diagnosed cancers. We have recalculated the percentage of gastric, colonic and pancreatic cancers over the total number of digestive cancers. In the previous version of the manuscript, the percentage was calculated based on the total number of cancers, which may have caused some confusion.

**Reviewer #3:** In this study, the authors use retrospective data collected at a single rapid diagnostic unit from years 2005-2013 of 791 patients presenting with involuntary weight loss above 5%. The cross-sectional visits are linked to diagnostic and mortality outcomes, allowing the authors to draw conclusions about IWL, disease, and mortality. The authors find, for example, that almost a quarter of patients with IWL were later diagnosed with cancer.

Thank you for the opportunity to read this interesting manuscript. I joined as a reviewer following the first set of revisions, and can see that both the reviewers and authors have already substantially improved the paper. I have just a few comments.

**Specific major comment #1**

1. Percentage weight lost. In the list of variables collected, is initial/presenting weight measured/asked? How is % lost calculated? If the variable is available, it should be presented in the descriptive tables. This is especially true in illnesses that occur more in men/women. For example, since women weigh less than men on average, and are more likely to present with psychiatric illness, the % weight lost would be higher in this category. This would be interesting to see.

**Response:** In the anamnesis, the physician asked the patient about the number of kilos lost in the last 12 months, and about the initial/presenting weight in order to be able to know if the loss was equal or superior to 5%. However, information regarding the initial/presenting weight was not always registered in the patient's clinical records. Weight loss percentage was not included as a study variable.

We agree that it would have been very interesting to have this information, but given the retrospective nature of the study, this was not possible.

**Specific major comment #2**

2. Brief question about “family support”. The phrase is referred to a few times in the manuscript. A brief phrase expanding what is meant by it (and why support is important in predicting RDU usage) would be helpful to the reader.

**Response:** One of the requirements for entering the RDU is the availability to attend additional visits and undergo tests on an outpatient basis in order to avoid hospital admission. Therefore, patients must be able to travel independently or otherwise have a primary caregiver who can accompany them.

**Specific major comment #3**

3. Previous research. While I wouldn’t usually ask an author to refer to my own work when writing a review, I think it could benefit the current manuscript. My co-authors and I are demographers and sociologists, so while the paper uses different survey and analytic methods, the findings could be useful for comparison with this manuscript and encourage cross-discipline dialogue. Though the papers differ--our paper, for example, used a nationally-representative survey that considered people who were diagnosed before retrospective survey, we didn’t exclude based on symptoms, and respondents didn’t need to present to a physician to confirm weight change—we also find evidence of the important role cancer plays in IWL.

Vierboom YC, Preston SH, Stokes A (2018) Patterns of weight change associated with
disease diagnosis in a national sample. PLoS ONE 13(11): e0207795. <https://doi.org/10.1371/journal.pone.0207795>

**Response**: The reviewer is totally justified in referring to her work as previous evidence of weight loss in a representative sample of the general adult population, according to their diagnosis with one of the six most prevalent pathologies either 0-1 years previously or 2+ years previously.

There are discrepancies in the literature about whether weight loss varies depending on the etiology. Although we did not observe differences in the average weight lost in the last 12 months between different etiological groups, the study corroborates previous reports stating that cancer could be associated with a higher probability of unintentional weight loss.

We have added this reference.

**Specific major comment #4**

4. Will there be statistical programming code available to readers, alongside the data? It’s becoming customary in my field to make not just the data but also the code that produced the analysis and tables publicly available, to encourage transparency and reproducibility (of course, may be different in the medical field).

**Response**: At the time we designed the study, we did not consider sharing patient data or statistical programming codes. However, if a researcher asks us for this information, we are happy to share it.
